# Retrospective Study of First Trimester Metrorrhagia: Pregnancy Follow-Up and Relationship with the Appearance of Gestational Complications

**DOI:** 10.3390/medicina59081370

**Published:** 2023-07-27

**Authors:** Laura Baños Cándenas, Daniel Abehsera Davó, Lucía Castaño Frías, Ernesto González Mesa

**Affiliations:** 1 Medicine School, Malaga University, 29071 Málaga, Spain; 2 Obstetrics and Gynecology Service, Virgen de la Victoria University Hospital, 29010 Málaga, Spain; 3 Obstetrics and Gynecology Service, HM Málaga Hospital, 29010 Málaga, Spain; 4 Obstetrics and Gynecology Service, Regional University Hospital of Malaga, 29011 Málaga, Spain; 5 Surgical Specialties, Biochemistry and Immunology Department, Malaga University, 29071 Málaga, Spain; 6 Biomedical Research Institute of Malaga (IBIMA) Research Group in Maternal-Fetal Medicine, Epigenetics, Women’s Diseases and Reproductive Health, 29071 Málaga, Spain

**Keywords:** first trimester bleeding, metrorrhagia of the first trimester, emergency room, gestational complications, preeclampsia, gestational diabetes, abortion, threatened preterm labor, prematurity

## Abstract

*Background and Objectives*: The purpose of this study was to describe and evaluate the bleeding that occurs during the first weeks of gestation and its implications throughout pregnancy. Secondarily, we assessed the associated complications in order to identify potential risk factors that could be used to select women at higher risk of adverse outcomes that could benefit from an early diagnosis and improved monitoring. *Materials and Methods*: We made a selection of all the women who consulted in the Emergency Department of the Hospital QuirónSalud in Malaga on 2015 presenting with first trimester metrorrhagia. We refer to first trimester metrorrhagia as that which occurs until week 12 + 6. Once these pregnant women were identified, we studied several variables not related to the gestation and some others associated with it and its natural course. *Results*: The average age of the patients assessed was 34.1. Associated gestational complications were metrorrhagia in the second trimester (6.3%), threatened preterm labor (7.4%), preeclampsia (2.5%), gestational diabetes (7.4%), late abortion (1.2%), and early postpartum hemorrhage (1.8%). We sought associations to assess possible risk factors, establishing an increased maternal age as an aggravating factor for the development of complications. We also studied gestational complications, finding a higher prevalence of them in older women, such as prematurity (33.11 vs. 34.48 years), gestational diabetes (33.11 vs. 36.06 years), and preeclampsia (33.25 vs. 35 years). *Conclusions*: Maternal age is a risk factor for first-trimester spontaneous miscarriage and for the development of complications of pregnancy. It is crucial to perform a correct screening of different pathologies throughout the pregnancy to anticipate potential complications.

## 1. Introduction

Metrorrhagia or first trimester bleeding is defined as bleeding before 20 weeks of gestation [1,2]. Bleeding of genital origin during the initial stages of pregnancy is a frequent problem (20–30%) and has been shown to be associated with increased risk of adverse pregnancy outcomes, such as preeclampsia, gestational diabetes (GD), preterm delivery, threatened preterm labor, and preterm premature rupture of the membranes (PPROM). Other studies also include small-for-gestational-age (SGA) fetal measurements, low birthweight, and fetal death, though evidence is unclear [3]. Bleeding in the early stages of pregnancy is of great concern to the patient. This is one of the most common reasons for consultation in emergency services [4]. The main causes of bleeding in the first trimester include voluntary termination of pregnancy, spontaneous abortion or miscarriage, ectopic pregnancy, gestational trophoblastic disease, and various non-obstetric causes (cervicitis, vaginitis, trauma, cervical cancer, polyps) [1].

The Spanish Society of Obstetrics and Gynecology (SEGO) defined pregnancy loss as “the expulsion or removal from its mother of an embryo or foetus weighing less than 500 g (approximately 22 completed weeks of pregnancy) or other absolutely non-viable product of pregnancy of any weight or gestational age, whether or not there is evidence of life or whether the abortion was spontaneous or induced” [5].

The World Health Organization (WHO) describes pregnancy loss as the expulsion of the embryo before 20 weeks of gestation, distinguishing between early pregnancy loss, before week 12, and late abortion, between week 12 and 20.

Early pregnancy loss is one that occurs before 12 weeks. Within early pregnancy loss, we can describe biochemical, preimplantation or preclinical abortion. These types of miscarriage occur prior to implantation and therefore, before the clinical and ultrasound appearance of pregnancy is established, so for their diagnosis it is sometimes necessary to determine the value of β-hCG [6].

We refer to late abortion as that which takes place after the 12th week of gestation [7].

There is no consensus on the terminology, so there are variations in the different definitions used.

It can be challenging to precisely establish a diagnosis of miscarriage, because very early pregnancy losses generally go unnoticed, being classified as simple menstrual delays or dysfunctional metrorrhagia.

The etiology of spontaneous abortion is usually unknown in most cases. Numerous factors, from both maternal and embryonic origin, are involved in its development, such as infectious or environmental factors, which constitute the etiology of the majority of spontaneous abortions [8].

Due to its high prevalence among the population, we studied visits to the emergency room over a year and followed up these pregnancies to assess whether a relation between first trimester vaginal bleeding and the development of further complications throughout the pregnancy could be established. Any potential pregnancy-related complication can be extremely distressing for both the patient and her family. For the gynecologist, it is important to identify symptoms related to certain complications that may arise during the first weeks of pregnancy, as well as their potential short and long-term complications, in order to reassure, advise, and support the couple at such a difficult time. [9,10].

Despite its high prevalence, first trimester bleeding should not be considered physiological, and it is advisable to carry out an adequate differential diagnosis to rule out any possible obstetric, gynecological, or systemic underlying pathology.

The aim of the study was to review the cases of bleeding that occur in the first weeks of gestation and their implications throughout pregnancy. Secondarily, we studied the associated complications and tried to identify possible risk factors that could be used to select those women at higher risk of adverse outcomes that could benefit from early diagnosis and improved follow-up.

## 2. Materials and Methods

For this study, we selected women who consulted the Emergency Department of the Hospital QuirónSalud in Málaga throughout the year 2015 for first trimester bleeding. We refer to metrorrhagia in the first trimester as that which occurs during weeks 1 through 12. Once the pregnant women were identified, we studied risk factors already present prior to the gestation, and others intrinsic to the pregnancy and its natural course.

In the following table (Table 1), we can see a summary of the variables collected in our study.

Among the characteristics of our pregnant women, we collected maternal age in years during the first visit to the emergency room.

For each patient, we recorded parity, which refers to the number of times that the patient has previously given birth. We divided our patients into two subgroups: primiparous and multiparous.

For descriptive purposes of the sample, we recorded the obstetric history of the patients, including previous spontaneous abortions and surgeries performed on the uterus.

We also collected the number of visits to the emergency department due to first trimester bleeding, the gestational age at the time of the first visit, and those cases that resulted in miscarriage.

Once the gestation reaches weeks 7 + 6 and 13 + 6 of amenorrhea, the first trimester analysis is performed, preferably between the 8th and 10th week. Within this analysis, the value of PAPP-A and β-hCG are used as biochemical markers to perform the combined screening of aneuploidies. The values obtained are expressed in multiples of the median (MoM).

Pregnancy-associated plasma protein A (PAPP-A) is secreted by the syncytiotrophoblast and its concentration increases during pregnancy. There are studies that have shown the relationship between low values of PAPP-A and β-hCG detected in the early stages of pregnancy and several complications of pregnancy.

The relationship between the length of the cervical canal and prematurity has been demonstrated. Ultrasound measurement of the cervical length can be useful to predict which pregnancies are at increased risk of preterm delivery. The shorter the cervical length, the greater the risk. In the second trimester, this parameter is measured by ultrasound to detect pregnant women who are more likely to give birth prematurely.

Moreover, in the second trimester, we perform the O’Sullivan test, consisting of an oral overload with 50 g of glucose, and the subsequent measurement of blood glucose levels in venous plasma 60 min after ingestion.

This determination is performed on every pregnant woman in the second trimester regardless of whether or not they have any risk factors. However, it will only be performed during the first trimester to those women with risk factors, such as maternal age over 35, obesity (BMI > 30), a history of gestational diabetes or carbohydrate intolerance, first-degree relatives with diabetes, and history of fetal macrosomia. It is considered pathological when values are ≥140 mg/dL or ≥7.8 mmol/L. In these cases, a further diagnostic confirmation test is carried out using an oral glucose overload with 100 g of glucose.

In our study, we only used the value in mg/dL obtained in the analysis of the second trimester. Even if performed in some cases, we did not take into account results obtained from the analysis of the first trimester.

Gestational diabetes (GD) is defined as the form of diabetes that is first detected during pregnancy regardless of the need for insulin treatment, the degree of metabolic disorder involved, or its persistence after the end of pregnancy. For its diagnosis, the O’Sullivan test is used (pathological when ≥140 mg/dL) and a further confirmation test with an oral glucose overload of 100 g. The oral glucose tolerance test (OGTT) reference values are ≥105– ≥190– ≥165– ≥145 (fasting, 1-h, 2-h, and 3-h post glucose intake, respectively). For diagnostic confirmation, two of four abnormal values are required. We included in our study patients diagnosed with gestational diabetes during pregnancy using these criteria.

During pregnancy, hypertensive disorder is diagnosed by elevated blood pressure (BP) (systolic ≥ 140 and/or diastolic ≥ 90 mm Hg), in two or more measurements separated by 6 h. We define proteinuria as the presence of ≥300 mg of protein in a 24-h urine sample. Preeclampsia is defined as hypertension that appears after 20 weeks of gestation accompanied by proteinuria.

Second trimester hemorrhage is defined as bleeding occurring after 12 + 6 weeks, regardless of the amount.

Late abortion is defined as that gestational loss above week 12, we collect those above 12 + 6 weeks.

Preterm premature rupture of membranes (PPROM) refers to membrane rupture before 37 weeks of gestation. PPROM is estimated to complicate 3% of pregnancies and accounts for approximately one third (33.3%) of all preterm deliveries. Rupture of membranes occurring near the limit of fetal viability are much less frequent (estimated 0.04%) [11].

We compiled preterm membrane ruptures and analyzed those occurring at the limit of viability and pre-viability.

We define postpartum hemorrhage as any bleeding that occurs during the first 24 h after birth. We reviewed the obstetric history on admission and collected all the patients who had heavy bleeding, regardless of whether it was resolved with conservative medical treatment or by performing a puerperal curettage.

## 3. Results

### 3.1. Population

The total number of visits to the emergency room was 9451, 1161 of which were consultations about first-trimester bleeding. Our final sample of study consisted of 696 patients. The average age of the patients studied was 34.1, the median was 34, and age ranged from 18 to 50 years of age.

### 3.2. Parity

The rate of primiparous women was 54% and the rate of multiparous women was 46%.

### 3.3. Obstetric History

Within the obstetric history, we collected previous miscarriages and uterine surgeries.

**Figure 1:**
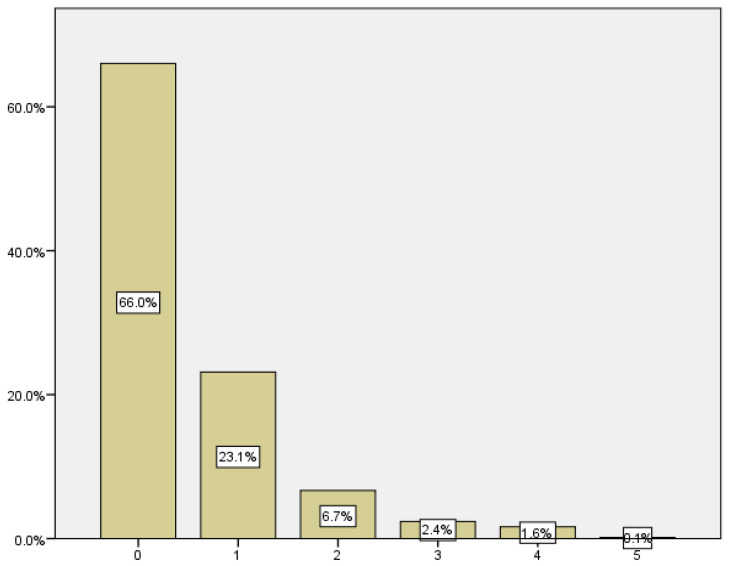
Distribution according to obstetric history.

The average number of previous miscarriages in our sample population was 0.51, with a standard deviation of 0.867. As we can see in Table 2 and Figure 1, in the sample, 66% of the women in our study had no history of previous miscarriages, while 44% had previous pregnancy losses. Among the latter, 23% had one previous spontaneous abortion, while the remaining 10.8% had more than one.

**Figure 2:**
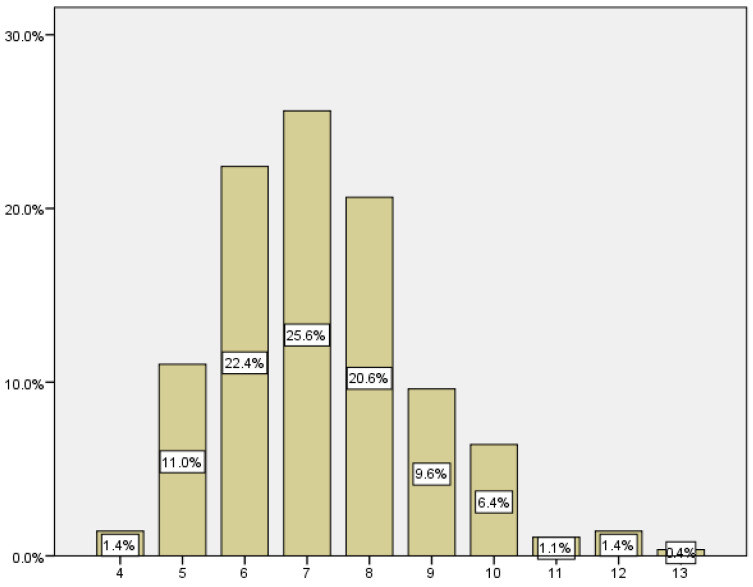
Distribution according to pregnancy loss gestational age.

We recorded the gestational age at which the pregnancy loss occurred by reviewing the emergency room visits and the outpatient follow-up. As we can see in the following table (Table 3), we found that 68.6% of the miscarriages occurred between 6 and 8 weeks of gestation, 22.4% in the 6th week, 25.6% in the 7th week, and 20.6% in the 8th week (Figure 2).

Regarding the existence of previous uterine surgery, we classified the patients according to whether or not they had previous surgeries.

Uterine surgeries reviewed included polypectomies by hysteroscopy, cesarean section, curettage, myomectomy, and conization for cervical dysplasia. We recorded data from a sample of 689 women, of whom 467 had not had uterine interventions prior to the current pregnancy, corresponding to 67.8% of the total. The remaining 222 patients (32.2%) had undergone some type of uterine intervention.

### 3.4. Number of Emergency Department Visits

Once the study population was selected, the number of times they visited the emergency department was reviewed as well as the reason for consultation. To establish the total number of visits to the emergency department, we recorded consultations for bleeding in the first trimester up to and including the 12th week of gestation (12 to 12 + 6 weeks). The remaining visits to the emergency department during pregnancy, as well as obstetric check-ups, were not taken into account for the study.

**Figure 3:**
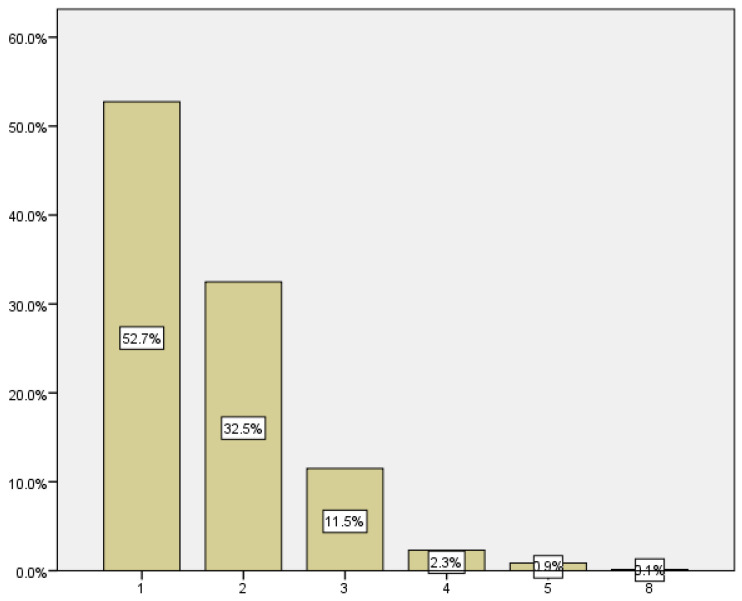
Distribution according to number of emergency visits.

In Table 4, we can see the summary of visits to the emergency room. The largest group of patients, who accounted for 52.7% and 32.5% of visits, attended the emergency room on one and two occasions, respectively; this group totals 85.2%. The remaining 14.8% of the patients visited the emergency department more than twice (Figure 3).

**Figure 4:**
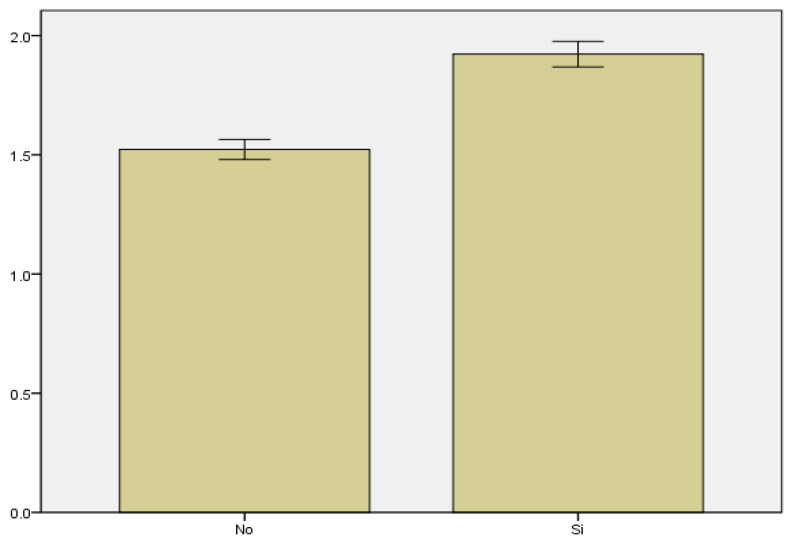
Distribution of first trimester miscarriages according to the number of visits to the emergency room.

We reviewed the number of times that patients consulted for first-trimester bleeding and assessed whether or not the pregnancy resulted in miscarriage. The average number of visits to the emergency room among patients who miscarried was 1.92 ± 0.923, compared to 1.52 ± 0.794 among those who did not (*p* < 0.0001) (Figure 4). This means that the patients who experienced pregnancy loss the most were those who had previously visited the emergency department on more occasions.

**Figure 5:**
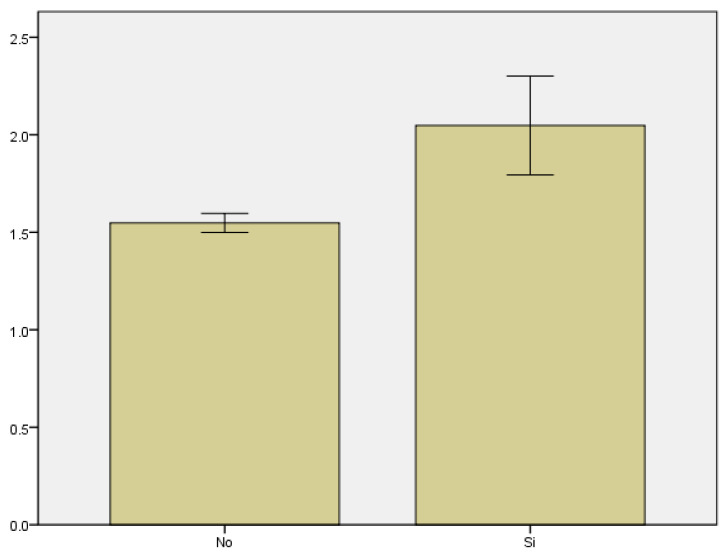
Distribution of threatened preterm labor according to the number of visits to the emergency room.

We combined the data collected on the number of visits to the emergency room before week 12 for first-trimester bleeding in pregnancies that did not result in miscarriage with the number of cases of threatened preterm labor. We found 21 cases of pregnancies that consulted for first trimester bleeding that were later diagnosed with threatened preterm labor compared to 263 who were not (Figure 5).

### 3.5. Gestational Age at the First Emergency Department Visit

**Figure 6:**
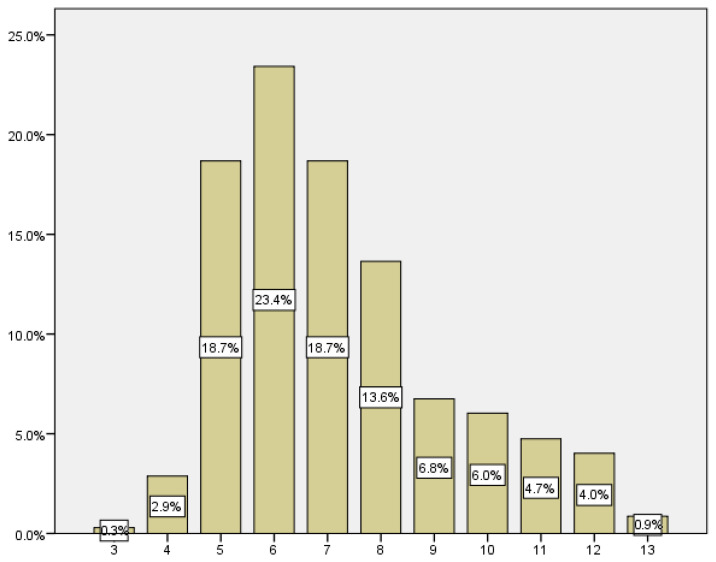
Distribution of gestational age at first visit to the emergency room.

The mean gestational age (number of weeks) at which the first consultation took place was 7.19 weeks, with a standard deviation of ±2.069. The highest percentage of women who consulted for first trimester bleeding (23.4%) did so in the 6th week of amenorrhea. The most part of the consultations occurred between the 5th and 8th week of gestation, constituting 74.4% of the consultations (Table 5) (Figure 6). 

### 3.6. Miscarriage

**Figure 7:**
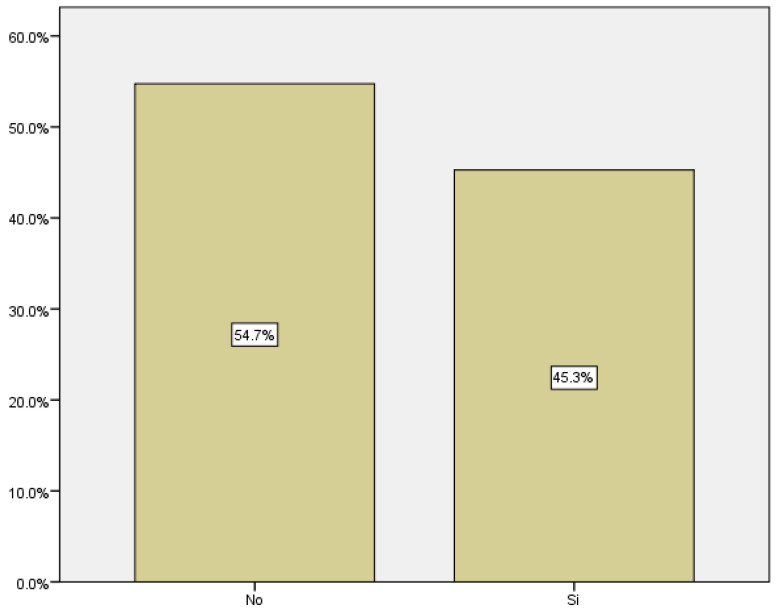
Distribution of miscarriages.

In our study, early pregnancy loss is defined as that occurring before 12 weeks, including 12 + 6 weeks. Of the 696 women who consulted for bleeding in the first trimester, 296 ended in miscarriage, which corresponds to 45.3% of the women who consulted for hemorrhage (Figure 7).

### 3.7. Second Trimester Bleeding

We considered second trimester metrorrhagia as vaginal bleeding occurring after the 13th week of gestation. Of the 268 pregnancies, 17 presented with second trimester bleeding, which corresponds to 6.3% of the total.

### 3.8. Cervical Length in Second Trimester

**Figure 8:**
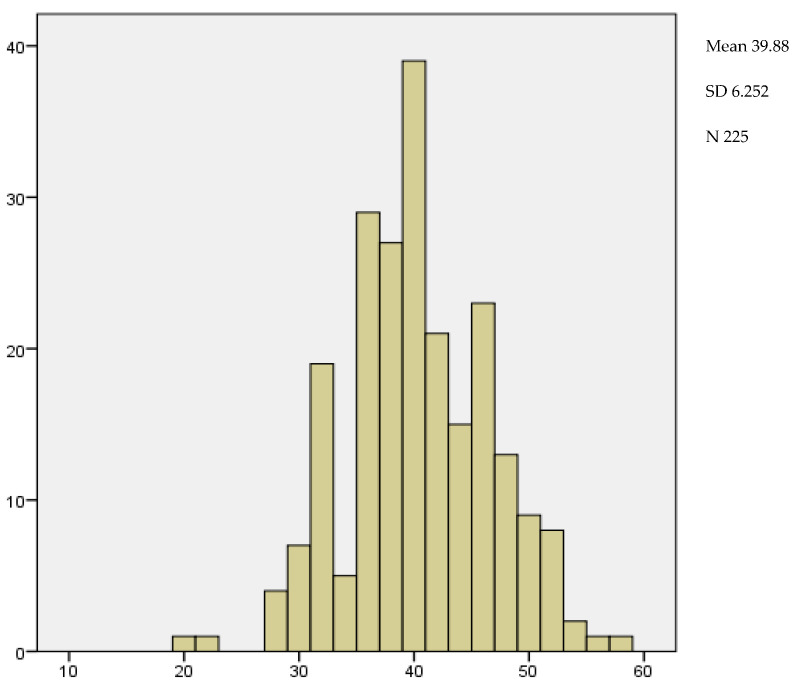
Distribution of cervical length.

The measurement of cervical length during the second trimester ultrasound is performed to establish a higher or lower risk of preterm delivery. The shorter the cervical length, the greater the likelihood of delivering before 37 weeks. The mean cervical length in our sample was 39.88 mm, with an SD of 6.25 mm. The smallest measurement recorded was 20 mm and the largest was 57 mm (Figure 8).

### 3.9. Preeclampsia

The calculation of the combined screening of the first trimester is based on several parameters: weeks of gestation according to the first day of the last menstrual period (LMP), crown–rump length (CRL), maternal age, nuchal translucency measured by ultrasound, and biochemical parameters such as PAPP-A and β-hCG expressed in multiples of the median (MoM). In our study, we recorded the value of PAPP-A in MoM.

The mean value of the PAPP-A was 1.0717, with a standard deviation (SD) of ±0.71. The lowest value was 0.21 and the highest was 5.89.

**Figure 9:**
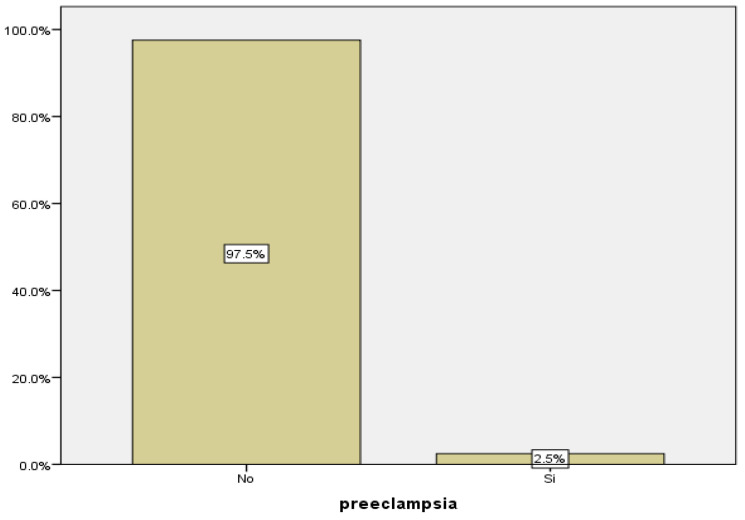
Distribution of cases of preeclampsia.

As previously stated, we define preeclampsia as hypertension that appears after 20 weeks of gestation accompanied by proteinuria. In our study, we recorded data from 243 pregnant women, 6 of whom were diagnosed with preeclampsia, corresponding to 2.5% of them (Figure 9).

We were able to establish a statistically significant relationship between blood levels of PAPP-A and the development of preeclampsia (*p* < 0.005). In the sample, 75% of women with preeclampsia presented with low PAPP-A levels during the first trimester analysis (Table 6).

Among all the women with low PAPP-A levels, only 11.1% had developed preeclampsia.

In summary, low PAPP-A levels are not associated with an increased risk of preeclampsia, but preeclampsia associates with low blood PAPP-A levels.

### 3.10. Prematurity

The gestational age of delivery was recorded in all pregnancies. We considered all pregnancies over 12 + 6 weeks, so this analysis may also include those that resulted in late abortions.

We consider late abortion to be gestational losses above week 12, up to and including week 12 + 6. In our sample, we have a total of 7 women who had a late abortion, which corresponds to 1.2%

**Figure 10:**
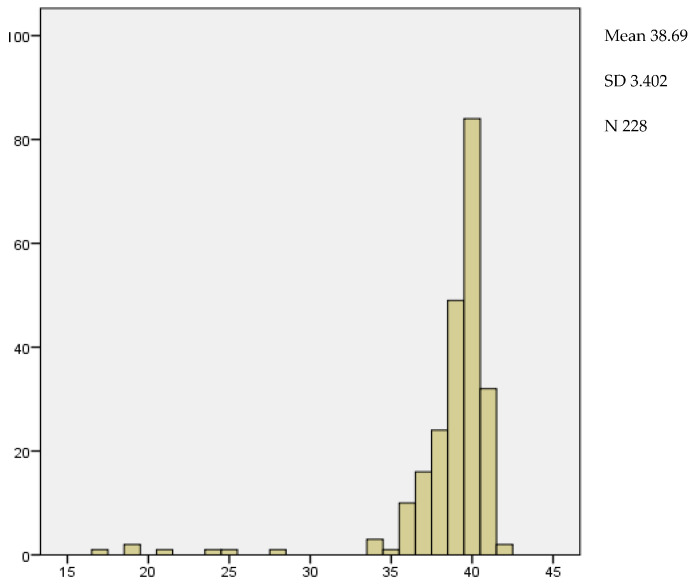
Average distribution according to the weeks of termination of pregnancy.

Gestational age at delivery ranged from 17 weeks, which corresponded to a late abortion, to 42 weeks, being 38.69 weeks with a SD of ±3.4 weeks mean gestational age at delivery (Figure 10).

**Figure 11:**
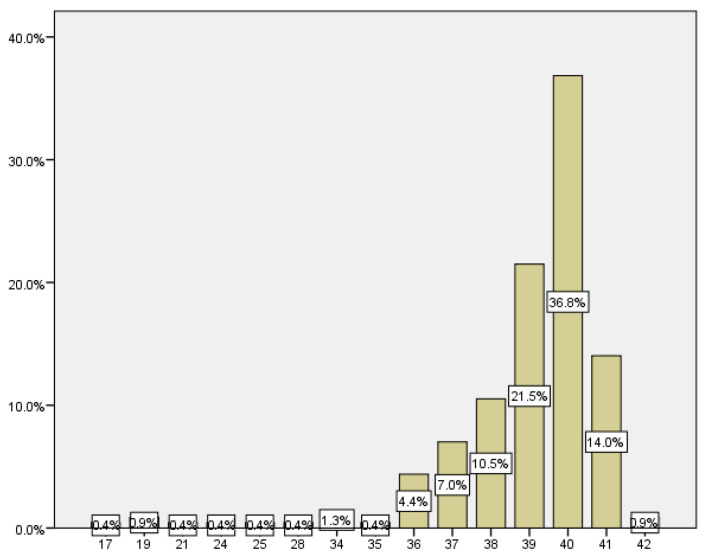
Distribution according to the weeks of termination of pregnancy.

The number of women who delivered before week 37 was 21, which corresponds to 9.2% of the total (Figure 11).

### 3.11. Preterm Premature Rupture of the Membranes (PPROM)

As for the cases of PPROM, considering those that occurred before 37 weeks, we recorded a rate of PPROM in our sample of 5.26%.

Among our results, we recorded four pre-viable preterm premature ruptures of membranes and one near the limit of fetal viability: two of them at 19 weeks, one at 17 weeks, one at 23 + 5 weeks, and another at 24 + 6 weeks. The percentage of previable preterm premature rupture of membranes in our study was 2.18%.

### 3.12. Gestational Diabetes

To screen for gestational diabetes, an oral glucose tolerance test using 50 g of glucose is performed in the second trimester, known as the O’Sullivan test. In those women with risk factors or history of gestational diabetes in previous pregnancies, this determination is done during the first trimester. In our study, we collected the results obtained in the second trimester, and considered the result pathological when glucose levels were ≥140 mg/dL. 

**Figure 12:**
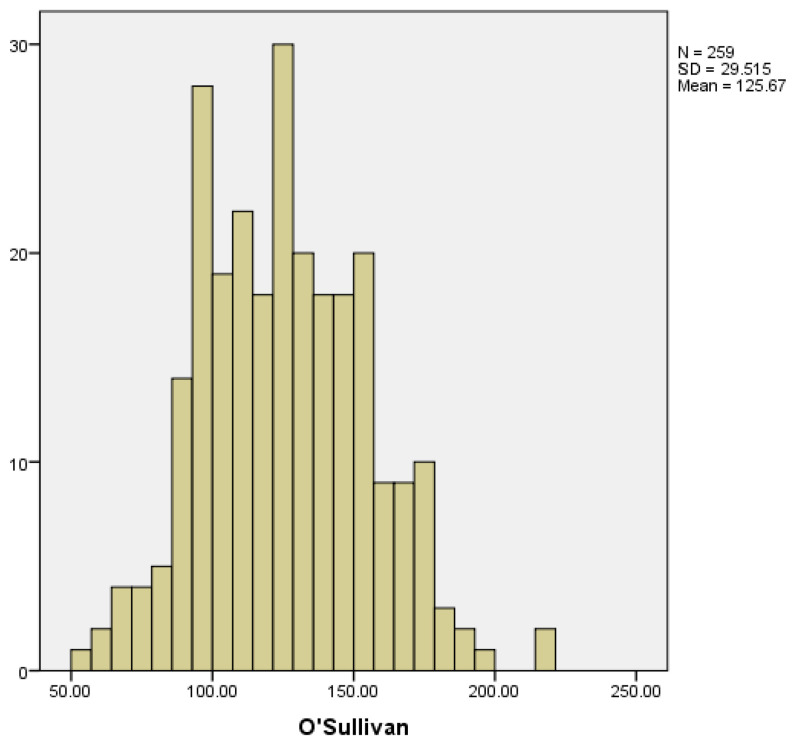
Distribution of O’Sullivan levels.

We collected data from 259 pregnant women. The mean glucose value obtained from the O’Sullivan test was 125.67 ± 29.51 mg/dL. The lowest and highest recorded values were 51 and 221 mg/dL, respectively (Figure 12).

**Figure 13:**
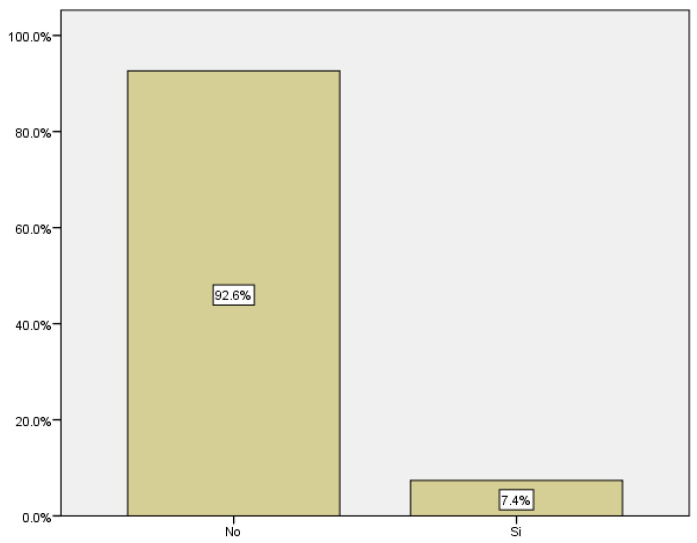
Distribution of cases of gestational diabetes.

We only took into account cases of gestational diabetes diagnosed during pregnancy; cases of diabetes diagnosed before were not included in the study. Among the women included in the study, 18 developed gestational diabetes, which corresponds to 7.4% of the total (Figure 13).

### 3.13. Early Postpartum Hemorrhage

We consider early postpartum hemorrhage to be that which occurs within 24 h after birth. Of the 228 pregnancies in which we registered deliveries, four of them had early postpartum hemorrhage, which corresponds to 1.8%.

We expanded the study by selecting the patients based on maternal age to assess whether maternal age relates to certain obstetric complications.

### 3.14. Threatened Preterm Labor

**Figure 14:**
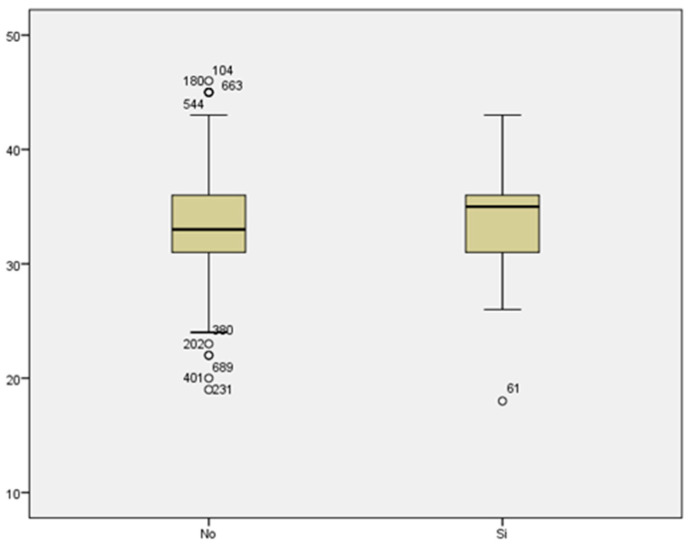
Boxplot distribution of threatened preterm labor cases in relation to maternal age.

We reviewed the cases of threatened preterm labor. We did not find a statistically significant relationship between maternal age and threatened preterm labor (33.40 ± 4.462 vs. 33.41 ± 5.335 years) (Figure 14).

### 3.15. Maternal Age

**Figure 15:**
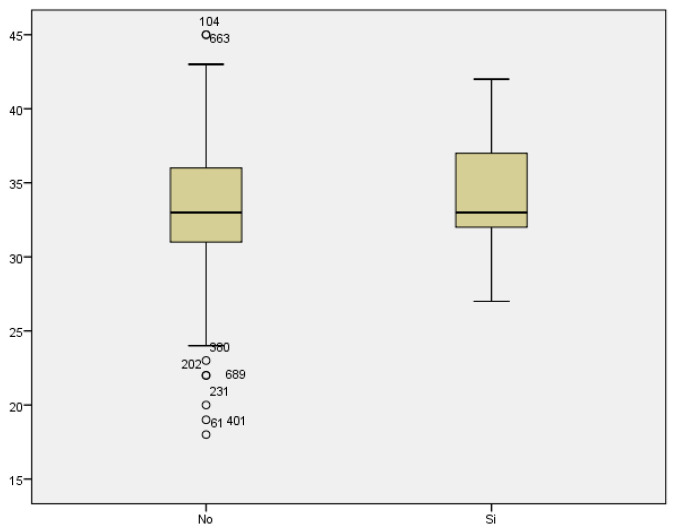
Boxplot distribution of cases of prematurity in relation to maternal age.

Regarding prematurity, we assessed maternal age in relation to having a preterm delivery. The mean maternal age of women who delivered before week 37 was 34.48 years, while it was 33.11 years for those who delivered at term (Figure 15). We found no statistically significant difference between both groups (*p* < 0.187).

**Figure 16:**
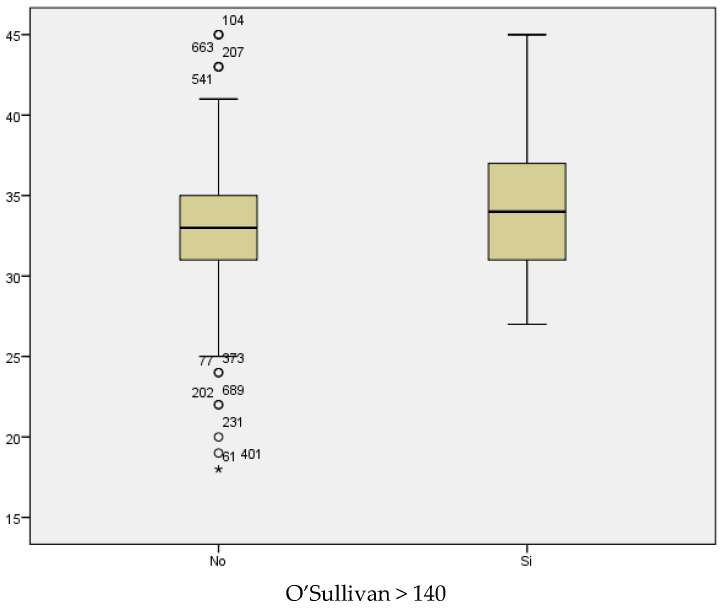
Boxplot distribution of pathological O’Sullivan cases in relation to maternal age.

If we analyze the results of the O’Sullivan test (using >140 mg/dL as the cut-off value) in relation to maternal age, we conclude that the mean maternal age for those women with test results within the normal range was 33.04; whilst the mean maternal age among women with pathological test results was 34.12 years (Figure 16). Therefore, the result seems to be influenced by maternal age, with higher prevalence of pathological test results with increased maternal age. However, statistical significance was not found (*p* < 0.069).

**Figure 17:**
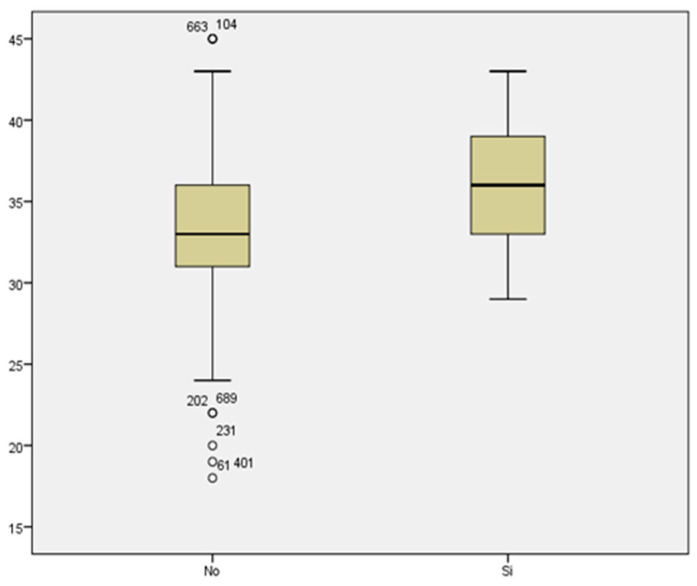
Boxplot distribution of cases of gestational diabetes in relation to maternal age.

Comparing the mean maternal age in women who developed gestational diabetes with respect to those who did not, we found a statistically significant difference, with *p* < 0.007. The mean maternal age of those who presented with GD was 36.06 years, compared to a mean age of 33.11 years of those who did not suffer from GD (Figure 17). Therefore, the prevalence of GD increased with maternal age.

**Figure 18:**
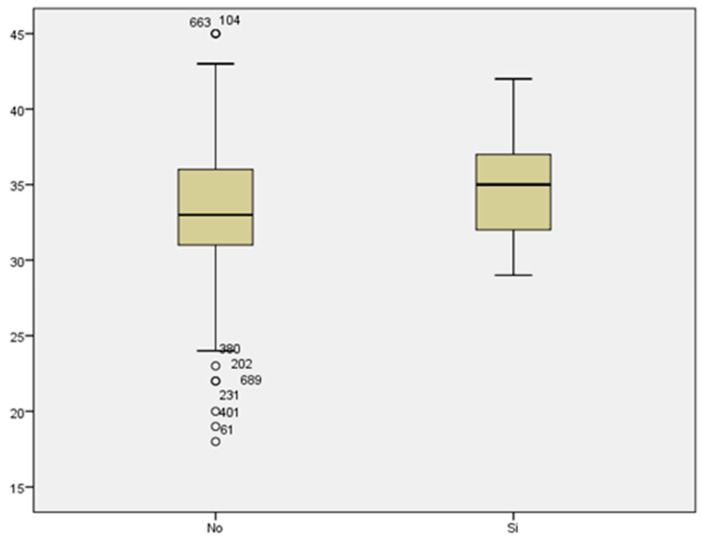
Boxplot distribution of cases of preeclampsia in relation to maternal age.

We did not find a statistically significant difference between the mean maternal age of women who developed preeclampsia and women who did not (35 and 33.25 years of age, respectively) with a value of *p* < 0.350 (Figure 18).

## 4. Discussion

Our study focuses mainly on the knowledge of bleeding in the first trimester and its implications throughout pregnancy.

Within the data reviewed in the patients who presented with bleeding in the first trimester, we collected different variables such as maternal age, obstetric history, number of previous abortions, and existence of previous gynecological surgery. Data on gestational follow-up and the appearance of associated obstetric pathology were also collected.

We selected the pregnant women who consulted for bleeding in the first trimester, we did not find a triggering cause of the bleeding.

In the following section, we describe each of the variables and results obtained in our study.

### 4.1. Obstetric History

In our sample, 44% of the pregnant women had a history of a previous pregnancy loss. This history increases the risk of subsequent miscarriages, thus the chances are estimated at approximately 16–20, 30, and 40%, depending on the history of 2, 3, or more pregnancy losses. Other authors find similar figures, with an increase in risk of miscarriage of 30% and 33% after the second and third miscarriage, respectively. For this reason, the most important predictor of recurrent pregnancy loss is a history of a previous pregnancy loss [12,13,14,15].

We reviewed the surgical history of the patients and found that the rate of previous uterine surgery was 32.2%. Of the patients with uterine interventions prior to the pregnancy, 46.39% experienced first trimester pregnancy loss. There was no difference in the results compared to those without previous surgical interventions performed on the uterus. In terms of the route of delivery, 43.24% of mothers gave birth by vaginal birth, while 56.7% had a cesarean section. We see that the caesarean section rate is somewhat higher than the rate observed the global population. This can be explained by the fact that the surgical history includes those patients with a previous cesarean section, so cesarean section is more frequent in this group [16].

However, we did not find statistical significance for any of the associations studied. There are no major complications or increased risk of threatened preterm labor, preeclampsia, late miscarriage, prematurity, or early postpartum hemorrhage in patients who underwent uterine surgery before pregnancy.

### 4.2. Number of Emergency Department Visits

In our study, we observed a relationship between the number of visits to the emergency department and the subsequent occurrence of a higher number of complications.

The complications with which we could establish a relationship were first-trimester pregnancy loss and threatened preterm labor.

Regarding the number of visits to the emergency department, a study on fetal growth patterns in pregnant women with first trimester bleeding found that bleeding lasting more than one day was associated with lower fetal birth weight [3].

Other studies in relation to low birth weight show similar results, with lower birth weight in those pregnant women who presented bleeding in the first weeks [17,18,19].

### 4.3. Gestational Age at the First Emergency Department Visit

The mean gestational age of consultation for first-trimester bleeding is 7.19 weeks. The gestational age with the most consultations was the 6th week, with 23.4% of the cases. Results published in a study on bleeding patterns and its predictive factors in the first trimester are in line with ours, the gestational age at which most consultations occur is between the 5th and 8th weeks, peaking between the 6th and 7th weeks [20]. 

A recent mortality study in the United States in 2020 reports that 80.9% of miscarriages occur below 9 weeks, and 93.1% before week 13 [21].

According to different studies, if the embryo develops and displays cardiac activity, the chances of pregnancy loss decrease drastically to 3–4%. An increased gestational age at which bleeding occurs for the first time is related to a better prognosis, thus 90 to 96% of pregnancies between the 7th and 11th weeks in which vaginal bleeding occurs and there is already embryonic cardiac activity will evolve. The higher the gestational age, the higher the pregnancy success rate [7,22].

**Figure 19:**
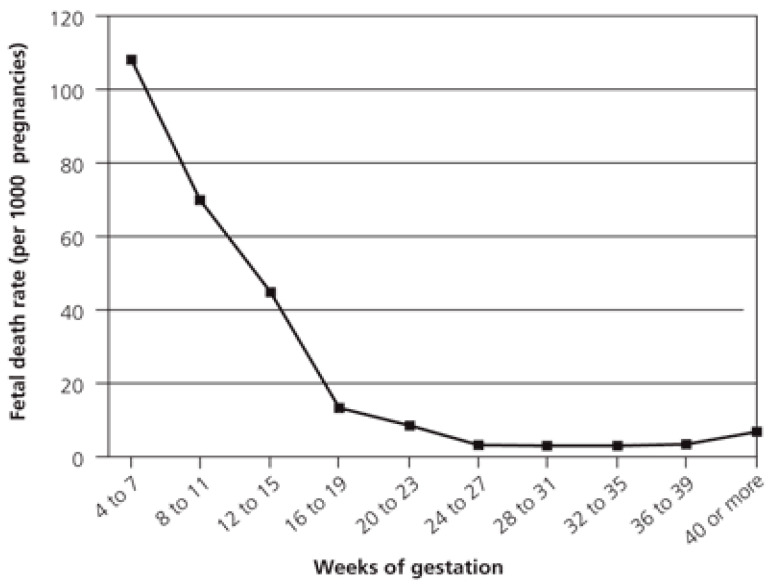
Distribution of miscarriages according to gestational age [23].

The following graph (Figure 19) shows how the prevalence of spontaneous abortions is higher in the first weeks of gestation, and how it decreases as gestational age increases.

Therefore, it would be advisable to pay special attention to the patients who consult for first trimester hemorrhage and who have a history of previous miscarriages.

The most frequent gestational age at which gestational losses occur is between the 6th and 8th week (68.6%). According to the literature, approximately 90–95% of miscarriages occur in the first trimester, most of them before the 8th week.

### 4.4. Miscarriage

Among all the pregnant women who consulted for first trimester bleeding, the miscarriage rate obtained was 45.3%. Comparing this result with the miscarriage rate in the general obstetric population, regardless of the presence or absence of bleeding, the miscarriage rate is approximately 12–15% [24].

Reviewing the literature on first trimester pregnancy losses and their prevalence, we found similar results to those obtained in our study. Approximately 50% of pregnancies that present with bleeding in the first weeks result in miscarriage.

### 4.5. Second Trimester Bleeding

Regarding second trimester bleeding, 6.3% of the pregnancies that reached the second trimester also presented with bleeding then. In the publications reviewed, we found a rate of bleeding in the second trimester of 3–4%, somewhat lower than that obtained in our study. This may be explained by the fact that our patients already had bleeding in the first trimester, so it may be more common for them to present with or maintain bleeding until in the second trimester [10,15,17].

### 4.6. Cervical Length in Second Trimester

We also reviewed the value of cervical length in the second trimester. The average value of our patients was 39.88 mm. According to the results obtained, therefore, it was not decreased, the cervical length in our patients was not diminished. Measurements above 30 mm are considered normal and those below 25 mm are associated with a greater risk of threatened preterm labor [25].

We compared the cervical length in those patients who presented APP with respect to those who did not present it and found a statistically significant difference. We found a relationship between the shorter cervical length and threatened preterm labor.

Multiple studies have previously demonstrated this relationship between the measurement of the cervical canal and subsequent presentation of threatened preterm labor and prematurity [25,26,27].

### 4.7. Preeclampsia

The rate of preeclampsia in our patients was 2.5%, a lower value than that estimated globally, approximately between 5 and 10%. According to the WHO, this rate is seven times higher in developing countries and in areas of greater prevalence of cardiovascular disease [28,29,30].

In our study, we did not find an increase in cases of preeclampsia. However, we observed that 75% of patients with preeclampsia presented low blood levels of PAPP-A in the laboratory tests performed on the first trimester.

We found studies showing similar results, in which they also observed a greater risk of preeclampsia, preterm labor, and low birth weight, in pregnant women with low levels of PAPP-A [31,32,33].

As previously stated, low levels of PAPP-A do not relate to an increased risk of preeclampsia, but preeclampsia is related to having low blood PAPP-A levels.

In our sample, we used a value of 0.6 MoM as a cuff-off for PAPPA-A, with values lower than 0.6 being considered low. Other authors used values of 0.4 and 0.2 MoM as the cuff-off, observing more striking statistically significant differences in terms of adverse outcomes in these two groups. The most severe cases, associated with intrauterine fetal death or placental abruption, presented for the most part values of PAPPA-A below 0.2 MoM [34].

For the above-mentioned reasons, the measurement of serum PAPP-A during the first trimester analysis may be useful to predict future adverse outcomes in pregnancy [35].

### 4.8. Prematurity

Regarding pre-term delivery, understood as that which takes place before the end of week 37 of gestation, we found a rate of 9.2% preterm births among our patients. 

According to data from the National Institute of Statistics, the prematurity rate represents 6.5–9% of all births and may increase to and reach 12.5% in reference centers. We compared the prematurity rate obtained in our study with that published by other work groups and found similar results, with a rate of pre-term birth around 10.24% [36,37].

We studied maternal age as a risk factor for prematurity, finding no statistically significant differences in the rate of prematurity with increasing maternal age.

We analyzed the data published by other authors on this matter and found disparate results. There are publications with similar results to ours, in which they did not find an increase in prematurity [38]. However, other authors have documented an increase in prematurity among pregnant women of advanced aged. This increase could be influenced by the greater degree of associated maternal pathology, which in turn could result in a premature delivery [39]. 

A recent study shows the same conclusions; it assessed the association between maternal age and prematurity and found no increase in maternal age among pregnancies that terminated spontaneously, while they demonstrated a rise in prematurity with increased maternal age among pregnancies that required a planned delivery due to associated medical complications. Therefore, there was a correlation between increased maternal age and prematurity but as a result of maternal medical conditions that required planned delivery [36]. 

### 4.9. Threatened Preterm Labor

Regarding threatened preterm labor, as previously stated, the data we obtained were in line with that previously published in other studies. We classified the patients according to maternal age and did not observe a statistically significant increase in the cases of threatened preterm labor with increasing maternal age. However, other studies found greater risk of threatened preterm labor, which resulted in increase rates of hospitalizations and the need for specific tocolytic treatment [38].

### 4.10. Gestational Diabetes

The rate of gestational diabetes in our sample (7.4%) is similar to that previously reported by other studies. The latest studies show rates ranging from 7 to 14%, with a significant increase in recent years [40]. However, other studies suggest rates somewhat higher than ours; for instance, it is estimated that 15% of pregnancies are affected by DG in Australia [12].

In line with the rest of our study, we classified women according to age in order to assess whether there is an association between increases in maternal age and the development of GD. In this case, we found that there is also a correlation between maternal age and GD, with increasing maternal age being a risk factor for GD.

### 4.11. Preterm Premature Rupture of the Membranes (PPROM)

Premature rupture of membranes occurs before the onset of labor, with subsequent loss of amniotic fluid. Most cases of PROMs occur at term; preterm PROM is uncommon and complicates 2–4% of all singleton pregnancies [41].

According to other authors, preterm premature rupture of membranes complicates approximately 3% of pregnancies [11,42]. 

In a study based on late pregnancy outcomes associated with first trimester vaginal bleeding (FTVB), compared to control group, the FTVB cases had more premature rupture of membranes (OR = 10). The incidence of premature rupture of membranes was 3.6% in the controls and 27.1% in the cases (RR = 10, *p* < 0.001) [43]. 

The rate of preterm premature rupture of membranes in our sample was 5.26% and accounted for 57.14% of all preterm deliveries. This rate is higher than that published in the general population.

Within these PPROMs, we have four pre-viable preterm premature rupture of membranes and one rupture near the limit of fetal viability, which represents 2.18% of all PPROM. This value is much higher than that reported by other studies, less than 1% [11].

As important data in the follow-up of these patients, it should be noted that all of them presented bleeding in the second trimester.

Therefore, closer monitoring of patients with FTVB and subsequent bleeding during the second trimester would be recommended.

The incidence in our study is somewhat higher than the global incidence in PPROM and much higher in pre-viable preterm premature rupture of membranes, finding similar data, even higher, than other authors.

### 4.12. Late Abortion

The rate of late miscarriage in pregnancies that presented bleeding in the first trimester was 1.2%. The rate of late abortions in gestations between the 13th and 19th weeks ranges from 1 to 5%, and the rate drops to 0.3% between weeks 20 and 27 [23]. In our study, there were seven late abortions between weeks 17 and 24. Therefore, the rate of miscarriage in our sample is similar to that published by other authors.

Possible causes of pregnancy loss in the second trimester due to maternal pathology include placental problems, thrombophilia, new onset of severe acute diseases, poorly controlled chronic disorders (diabetes, hypertension, etc.), immunological factors (lupus, antiphospholipid syndrome, etc.), infections, cervical insufficiency, and anatomical abnormalities [21].

### 4.13. Early Postpartum Haemorrhage

Regarding the data collected on early postpartum hemorrhage, only four women in our study developed this complication (1.8%), only one of them was older than 35 years. Therefore, we found no relationship between maternal age and increased frequency of early postpartum hemorrhage. We found one study whose results were in agreement with ours, and also failed to demonstrate an increase in early postpartum hemorrhage with increasing maternal age [44]. However, most studies do find a clear rise in the incidence of this complication with increasing maternal age [45,46,47,48,49,50,51,52].

In addition, many authors previously studied the relationship between increased maternal age and poor perinatal outcomes [47], and found in their analysis a statistically significant increase in the prevalence of GD, hypertensive disorders, induction of labor, and cesarean sections in women over 35 years of age [53].

As has been well-established, advanced maternal age increases the risk of several pregnancy-related complications. Consequently, we must emphasize the need to screen for GD and maintain strict control of blood pressure levels in all pregnant women, especially among the older ones.

## 5. Strengths and Limitations

Our study had several strengths. First, the sample of our study was constituted by all the patients that visited the emergency department of our hospital throughout a year, and their reasons for consultation were analyzed. Week 12 + 6 was established as the time limit for vaginal bleeding to be considered as first-trimester metrorrhagia, which allows us to establish clear inclusion and exclusion criteria.

Second, we have an acceptable sample size with a total of 696 pregnant women. Third, we carried out of a comprehensive assessment of each patient, reviewing previous medical and obstetric-gynecological history (abortions, uterine surgeries), current gestational data (first trimester screening and analytical values, cervical length, history of obstetric complications), and data regarding the delivery and the newborn (weeks of gestation, mode of delivery, newborn weight, Apgar test value, birth weight, etc.).

However, this study also has some limitations. As a first limitation, this is a retrospective study, therefore the data we used were limited to the information included in the digitized clinical history. Secondly, our study is solely based on visits to the emergency department of Hospital QuironSalud Málaga, and therefore the follow-up of the gestation and the subsequent analysis of obstetric history was performed in this same center. In consequence, data regarding patients with prenatal care at different centers were missing. We were able to retrieve some of the data lost by external monitoring from the collection of medical histories from the Andalusian Health System.

Another limitation was the absence of a control group with which to compare the results and assess the rate of complications among women who did not present with vaginal bleeding during the first trimester. Due to the high prevalence of first-trimester bleeding, and its potential implications for the pregnancy, it would be interesting to further continue the study and perform it prospectively, allowing for the establishment and control of potential risk factors that may lead to poor gestational outcomes.

## 6. Conclusions

In view of the results obtained in our study, which are in line with those previously observed by others, among the complications studied, we can establish a positive correlation between bleeding in the first trimester and two of them, miscarriage and pre-viable preterm premature rupture of membranes. However, we cannot establish a positive correlation between FTVB and the increased prevalence of various comorbidities and associated complications.

Although we found no association between the increase in gestational complications in women who presented with bleeding in the first trimester, in our study, we did observe a relationship between the appearance of complications with increasing maternal age. The increased risk of gestational outcomes in women with advanced age highlights the need for close follow-up, early detection, and management of medical complications.

## Figures and Tables

**Table 1:** Variable summary.

Variable Name
Maternal age
Parity
Number of emergency room visits
Gestational age at first visit
Previous uterine surgery
History of previous miscarriages
First trimester miscarriage
Weeks of miscarriage
Risk of trisomy 21 or Down Syndrome
Risk of trisomy 18 or Edwards Syndrome
PAPP-A
BHCG
TSH
Cervical length
Second trimester bleeding
Threatened preterm labor
Preterm premature rupture of the membranes
O’Sullivan value in the second trimester
Gestational diabetes
Preeclampsia
Gestational age at delivery
Way of delivery
Newborn weight
Venous pH
Apgar 1
Apgar 5
Early postpartum hemorrhage

**Table 2:** Obstetric history, number of previous miscarriages.

Number of Previous Miscarriages	Rate	Incidence
0	445	66.0
1	156	23.1
2	45	6.7
3	16	2.4
4	11	1.6
5	1	0.1
Total	674	100

**Table 3:** Gestational age at which pregnancy loss occurs.

Gestational Age	Rate	Incidence
4	4	1.4
5	31	11.0
6	63	22.4
7	72	25.6
8	58	20.6
9	27	9.6
10	18	6.4
11	3	1.1
12	4	1.4
13	1	0.4
Total	281	100

**Table 4:** Emergency visits.

Emergency Visits	Rate	Incidence
1	367	52.7
2	226	32.5
3	80	11.5
4	16	2.3
5	6	09
8	1	0.1
Total	696	100

**Table 5:** Gestational age at the first visit.

Gestational Age First Visit	Rate	Incidence
3	2	0.3
4	20	2.9
5	130	18.7
6	163	23.4
7	130	18.7
8	95	13.6
9	47	6.8
10	42	6.0
11	33	4.7
12	28	4.0
13	6	0.9
Total	696	100

**Table 6:** Relationship between preeclampsia and low PAPP-A levels.

			Preeclampsia		Total
			No	Yes	
Low PAPP-A	Yes	Count	24	3	27
		% with low PAPP-A levels	88.9%	11.1%	100.0%
	No	Count	106	1	107
		% with low PAPP-A levels	99.1%	0.9%	100.0%
Total		Count	130	4	134
		% with low PAPP-A levels	97.0%	3.0%	100.0%

## Data Availability

Not applicable.
